# Polymorphism in drug resistance genes dihydrofolate reductase and dihydropteroate synthase in *Plasmodium falciparum* in some states of India

**DOI:** 10.1186/s13071-015-1080-2

**Published:** 2015-09-17

**Authors:** Divya Sharma, Manila Lather, Prashant K. Mallick, Tridibes Adak, Amita S. Dang, Neena Valecha, Om P. Singh

**Affiliations:** National Institute of Malaria Research, Sector 8, Dwarka, Delhi-110077 India; Centre for Medical Biotechnology, Maharshi Dayanand University, Rohtak, 124001 India

**Keywords:** Plasmodium falciparum, Sulfadoxine-pyrimethamine, pfdhfr, pfdhps, India

## Abstract

**Background:**

Sulfadoxine-pyrimethamine (SP) combination drug is currently being used in India for the treatment of *Plasmodium falciparum* as partner drug in artemisinin-based combination therapy (ACT). Resistance to sulfadoxine and pyrimethamine in *P. falciparum* is linked with mutations in dihydropteroate synthase (*pfdhps*) and dihydrofolate reductase (*pfdhfr*) genes respectively. This study was undertaken to estimate the prevalence of such mutations in *pfdhfr* and *pfdhps* genes in four states of India.

**Methods:**

*Plasmodium falciparum* isolates were collected from two states of India with high malaria incidence i.e., Jharkhand and Odisha and two states with low malaria incidence i.e., Andhra Pradesh and Uttar Pradesh between years 2006 to 2012. Part of sulfadoxine-pyrimethamine (SP) drug resistance genes, *pfdhfr* and *pfdhps* were PCR-amplified, sequenced and analyzed.

**Results:**

A total of 217 confirmed *P. falciparum* isolates were sequenced for both *Pfdhfr* and *pfdhps* gene. Two *pfdhfr* mutations 59R and 108N were most common mutations prevalent in all localities in 77 % of isolates. Additionally, I164L was found in Odisha and Jharkhand only (4/70 and 8/84, respectively). Another mutation 51I was found in Odisha only (3/70). The *pfdhps* mutations 436A, 437G, 540E and 581G were found in Jharkhand and Odisha only in 13, 26, 14 and 13 % isolates respectively, and was absent in Uttar Pradesh and Andhra Pradesh. Combined together for *pfdhps* and *pfdhfr* locus, triple, quadruple, quintuple and sextuple mutations were present in Jharkhand and Odisha while absent in Uttar Pradesh and Andhra Pradesh.

**Conclusion:**

While only double mutants of *pfdhfr* was present in low transmission area (Uttar Pradesh and Andhra Pradesh) with total absence of *pfdhps* mutants, up to sextuple mutations were present in high transmission areas (Odisha and Jharkhand) for both the genes combined. Presence of multiple mutations in *pfdhfr* and *pfdhps* genes linked to SP resistance in high transmission area may lead to fixation of multiple mutations in presence of high drug pressure and high recombination rate.

**Electronic supplementary material:**

The online version of this article (doi:10.1186/s13071-015-1080-2) contains supplementary material, which is available to authorized users.

## Background

Malaria is one of the major health problems in tropical and subtropical countries. One of the greatest challenges to malaria treatment is the development and spread of resistance in parasites especially in *Plasmodium falciparum* which threaten the usable lifespan of even artemisinin-based combination therapies, affecting both the artemisinin component and the partner medicine [1]. India has evidenced resistant parasite especially *P. falciparum* against all available conventional antimalarials like chloroquine (CQ) and sulfadoxine-pyrimethamine (SP) [2]. A decade long use of artemisinin-based combination therapy (ACT) had been proved a hallmark anti-malarial therapy for all the malaria endemic countries [3]. The reports of chloroquine resistance in *P. falciparum* in early 1980s lead to introduction of SP as a second line antimalarial drug in CQ-resistant areas of India [4]. Sulfadoxine and pyrimethamine acts as a synergistic combination and was used as long acting partner antimalarial drug in ACT in South Asia, Middle East and South America [3]. Since 2005, Indian antimalarial drug policy has introduced artesunate with SP as ACT in place of SP in high malaria endemic areas, and later in 2010, this treatment became the recommended first line treatment throughout India [5, 6]. Further, since 2013, prevalence of resistant genotype of falciparum against this partner drug SP, led to introduction of artemether-lumefantrine as antimalarial therapy for northeastern part of India [7, 8].

The synergistic combination of the sulfadoxine and pyrimethamine inhibits dihydropteroate synthase (*dhps*) and dihydrofolate reductase (*dhfr*) enzymes respectively in the folate-pathway of parasite [9]. The development of resistance against SP emerges with a single point mutation in the parasite *dhfr* and *dhps* gene, which further augments with stepwise addition of mutations [10–12]. Resistance to pyrimethamine is primarily conferred by a point mutation at codon 108 and augmented by mutations at codon 16, 51, 59 and 164 of *Plasmodium falciparum dhfr* (*pfdhfr*) gene [10, 12, 13]. Similarly, point mutation at codon 436 or 437 in *Plasmodium falciparum dhps* (*pfdhps*) gene may initiate the resistance and followed by mutations at codon 540, 581 and 613, which are considered for augmentation of sulfadoxine resistance [11, 13, 14].

A single mutation in *pfdhfr* or *pfdhps* gene is not enough to cause treatment failure and multiple mutation combinations in these two genes were associated with failure of SP as anti-malarial therapy [15, 16]. Various parts of India reported single, double, triple and quadruple mutant *pfdhfr* gene [17–22]. However, double mutation at codon 59 and 108 in *pfdhfr* gene was predominant throughout India [17–22]. Triple mutant *pfdhfr* gene indicating high level of antifolate resistance was observed in India from northeast states, Car Nicobar island and Odisha [7, 15, 20, 22, 23]. Highly resistant quadruple mutant allele was observed in high and low frequencies from Car Nicobar island and northeastern parts of India respectively [19, 20, 22, 24, 25]. Wild-type allele in *pfdhps* gene was predominant in all geographic regions of India except Andaman and Nicobar island, where lower frequency of mutations in *pfdhps* gene was observed in comparison to *pfdhfr* gene*,* which supports that mutations first emerged in *pfdhfr* and then occurs in *pfdhps* gene [23]. Single mutation at codon 437 was observed in low frequency from Assam, Odisha, Madhya Pradesh and Uttar Pradesh [21, 23, 26]. Further, double and triple mutation including mutation at codon 437 was also observed in low frequencies from Madhya Pradesh, northeast and Odisha [20, 26, 27]. However, recent studies from northeastern part of India showed increased number of key mutation at codon 437 included in triple and quadruple mutations in *pfdhps* gene [7, 20, 22].

Development of resistance against ACT is currently a major threat and *P. falciparum* bearing resistance against its partner drug (here, SP) may lead to ACT failure [3]. The reports of widespread resistance against SP generate concern about long-term effectiveness of ACT in India [5, 20]. A recent study reported significant reduction in efficacy of SP treatment from northeastern areas of India, which is considered as a gateway for invasion of drug resistant parasite from Southeast Asia to India [7, 20, 22]. Thus, routine molecular surveillance of SP resistance markers is essential in malaria endemic regions, which will help in formulating an effective malaria treatment strategy. Here, we attempted to determine the changes in the frequencies of *dhfr* and *dhps* mutations in *P. falciparum* isolates from four states of India (Jharkhand, Odisha, Andhra Pradesh and Uttar Pradesh) to assess the level of SP resistance.

## Methods

### Study population and blood sample collection

Finger prick blood-spots (*n* = 217) were collected on Whatman 3MM filter paper from all microscopically-confirmed *P. falciparum* positive patients. This study has been approved by the Institutional Ethics Committee (IEC), and the Scientific Advisory Committee (SAC) of National Institute of Malaria Research. All isolates were collected between years 2006–2012. The study included patients with symptoms of uncomplicated malaria, visiting Primary Health Centre (PHC) situated in the six districts of the four different states of India, i.e., Jharkhand, Odisha, Andhra Pradesh and Uttar Pradesh which are described below. Artemesinin-based combination therapy was implemented as the first line of antimalarials drug for treatment of *P. falciparum* in some districts of Andhra Pradesh, Jharkhand and Orissa since 2008 and in Uttar Pradesh since 2010. Samples were collected from following sites:

1. *Jharkhand:* Microscopically confirmed *P. falciparum* samples were collected from villages under PHC Tamar, district Ranchi (23° 23' N latitude and 85° 23' E longitude) and East Singhbhum PHC (23° 01' N latitude and 86° 54' E longitude). The Annual Parasite Incidence (API, i.e., ‰ malaria case in a year) of Jharkhand is ≥5 [28, 29]. Samples (*n* = 84) were collected between year 2006 and 2011 during which proportion of *P. falciparum* malaria has increased from 24 % (*n* = 48388) to 44 % (*n* = 70302) in Jharkhand state [30].
2. *Odisha:* Samples were collected from villages under Rayagada PHC (19° 09' N latitude and 83° 27' E longitude) and Rourkela PHC (22° 25' N latitude and 85° 00' E longitude) of Odisha state are belonging to hyper endemic areas show a higher level of drug resistance and intense perennial malaria transmission. API of Odisha is ≥5 [28, 29]. Odisha alone contributed approximately 25 % of the total malaria cases and 45 % of total falciparum malaria cases reported in the country during recent years (2008–2012). All isolates (*n* = 70) collected from this site between years 2008, 2010–2012 during which proportion of *P. falciparum* malaria increased from 87 % (*n* = 329631) to 93 % (*n* = 244503) [30].
3. *Andhra Pradesh:* Samples were collected from PHC Salur, district Vizianagaram (18° 53' N latitude and 83° 21' E longitude) which is located in the northern part of the Godavari district which is dominated by tribal populations. Malaria endemicity is low, with annual transmission and API of Andhra Pradesh is ≥2 [29]. Samples (*n* = 32) were collection from this site during year 2011. During this period, *P. falciparum* was the predominant malaria spices in Andhra Pradesh and accounting for 70 % (*n* = 24089) of total reported malaria infection [30].
4. *Uttar Pradesh:* Samples were collected from PHC Razapur, district Ghaziabad (28° 40' N latitude and 77° 28' E longitude). Ghaziabad is an industrial area, located in between three river named Hindon, Ganga and Yamuna. Malaria endemicity is low with seasonal transmission and *P. vivax* is the predominant malaria species in this region. API of Ghaziabad is ≥2 [29]. Sample (*n* = 31) were collected from this site during years 2011 and 2012; during that time only 3.25 % (*n* = 1857) and 1.56 % (*n* = 740) of total malaria burden was attributed to *P. falciparum* in same years respectively [30].

### DNA isolation and molecular diagnosis

Genomic DNA was extracted from dried filter paper blood spots using QIAmp Blood mini kit (Qiagen, Krefeld, Germany) as per the manufacture’s instruction. A PCR diagnosis was performed to confirm the presence of *P. falciparum* infection and rule out any mixed species infection as described earlier [31].

### SNP’s genotyping in *pfdhfr* and *pfdhps* genes

#### PCR amplification of *pfdhfr* gene

A 720 base pair fragment of *pfdhfr* gene was amplified as described earlier [18] and nested PCR was performed to amplify 648-bp fragment covering various single nucleotide polymorphism (SNP) A16V, N51I, C59R, S108N and I164L correlated with pyrimethamine resistance. Primary PCR (25 μL) reaction contained 4 μl DNA template, 200 μM of dNTPs, 1x PCR buffer, 0.30 μM of primers AMP-1 F and AMP-2R, and 2.5 U Taq polymerase (Sigma, India). The cycling parameters used were as follows: an initial denaturation at 94 °C for 3 min, denaturation at 94 °C for 30 s, annealing at 45 °C for 45 s, and extension at 72 °C for 45 s, for 45 cycles followed by an extension step at 72 °C for 5 min. Nested PCR was performed using 2.5 μl templates from first round PCR product, 0.30 μM of primers M1 and M5, 200 μM of each dNTP, 1x PCR buffer, and 1 U of Taq polymerase in a 25 μl reaction. The PCR was carried at 94 °C for 3 min, followed by 35 cycles at 94 °C for 1 min, 45 °C for 1 min, 72 °C for 1 min and finally 72 °C for 10 min. The PCR amplicon was visualized in 2 % Agarose gel. The list of primers used for PCR-amplification is provided in Table 1.

**Table 1:** List of primers used in the study

Name of primers	Sequence (5’- 3’)	Expected size	References
AMP-1 F	TTTATATTTTCTCCTTTTTA	720 bp	[18]
AMP-2R	CATTTTATTATTCGTTTTCT		
M1	TTTATGATGGAACAAGTCTGC	648 bp	[38]
M5	AGTATATACATCGCTAACAGA		
M3717F	CCATTCCTCATGTGTATACAACAC	1287 bp	[18]
186R	GTTTAATCACATGTTTGCACTTTC		
Rc	GGTATTTTTGTTGAACCTAAACG	728 bp	[38]
Rd	ATCCAATTGTGTGATTTGTCCAC		

### PCR amplification of *pfdhps* gene

A nested PCR assay was performed to amplify 728-bp fragment of the *pfdhps* gene covering SNP’s S436A, A437G, K540E, A581G and A613S known to be associated with sulfadoxine resistance as described earlier [18]. Primary PCR reaction of 25 μL was prepared consisting 4 μl DNA template, 200 μM of each dNTP, 1x PCR buffer, 0.30 μM of primers M3717F and 186R and 2.5 U Taq polymerase (Sigma, India). The cycling parameters used were as follows: an initial denaturation at 94 °C for 5 min, denaturation at 94 °C for 30 s, annealing at 55 °C for 45 s, and extension at 72 °C for 90 s, for 45 cycles followed by an extension step at 72 °C for 10 min. Nested PCR was performed using 2.5 μl templates from first round PCR product, 0.30 μM forward primer (Rc) and 0.50 μM reverse primer (Rd), 200 μM of each dNTP, 1x PCR buffer, and 1 U of Taq in a 25 μl reaction. The PCR was carried at 94 °C for 4 min, followed by 30 cycles at 94 °C for 30 s, 50 °C for 40 s, 72 °C for 1 min and finally 72 °C for 10 min. The PCR amplicon was visualized in 2 % Agarose gel. The list of primers used for PCR-amplification is provided in Table 1.

### DNA sequencing and analysis

All successful nested PCR amplicons were purified using MinElute PCR Purification Kit (Qiagen, Krefeld, Germany) and subjected to DNA sequencing using Big-Dye Terminator Kit version 3.1 (Applied Biosystems, Foster, USA). Sequencing was performed on both strand of DNA to confirm SNP's. The chromatogram was manually edited in Finch TV and mixed bases were carefully scored. Sequences obtained were aligned in software MEGA version 5 [32] using ClustalW implemented in the program with the wild type sequence obtained from GenBank with Accession number J03028.1 and PFU07706 for *pfdhfr* and *pfdhps* respectively. DNA sequences obtained were submitted to GenBank (accession numbers KP30040 – KP300256 for *pfdhfr* and KP300257 – KP300473 for *pfdhps* gene). The genetic diversity parameters such as haplotype diversity and two measures of nucleotide diversity; θ_w_ and π were estimated in software DnaSP version 5.10.01. The estimation of θ_w_ and π is based on the number of segregating sites and mean number of pairwise nucleotide differences respectively. To test the neutrality in molecular evolution of *pfdhfr* and *pfdhps* gene, Tajima's D was calculated based on the normalized discrepancy between θ_w_ and π. Other measures of neutrality such as Fu and Li's D* and Fu and Li's F* were also evaluated.

### Sulfadoxine-Pyrimethamine resistance genotypes

Mutations at both genes were concatenated to form combined mutant genotypes, which in turn provide information about various levels of clinical resistance to SP treatment. In an earlier study [18], the combined mutant genotypes were categorized for following types of clinical resistance; category "S/RI" with single or double mutation in combined genotype infers early emergence of SP resistance; category "RI" with triple mutation in combined genotype suggests low level of SP-resistance; category "RI/RII" with triple mutation in *pfdhfr* gene suggests initiation of high level SP-resistance; category "RII/RIII" with quintuple mutations in combined genotype suggests higher level of SP-resistance; category "RIII" with sextuple mutations in combined genotype suggests total failure of SP treatment.

## Results

### Mutation analysis of *pfdhfr* and *pfdhps* genes

All 217 isolates were successfully sequenced for *pfdhfr* and *pfdhps* genes covering codon positions 16, 51, 59,108 and 164 of *pfdhfr* gene and codons 436, 437,540,581 and 613 of *pfdhps* gene. The distribution of amino acid substitution at various codon positions for *pfdhfr* and *pfdhps* genes are shown in Tables 2 and 3 respectively In total, sequencing results showed pure alleles at all the codons in *n* = 38 (17.51 %) isolates. No mutant alleles were detected at codon 16 in *pfdhfr* gene and at codon 613 in *pfdhps* gene. In *pfdhfr* gene, majority of isolates were observed with pure mutant alleles 59**R** (*n* = 117, 54 %) and 108**N** (*n* = 139, 64 %) and mixed mutant allele 59R* was observed in 23.04 % (*n* = 50) and 108 N* in 18.43 % (*n* = 40) isolates. However, pure mutant alleles 51**I** (*n* = 2, 0.92 %) and 164 **L** (*n* = 7, 3.22 %) were less prominent at codons 51 and 164 respectively. Mixed mutant alleles were observed in 0.46 % (*n* = 1) and 2.30 % (*n* = 5) at codon 51 and 164 respectively. Year-wise breakup of *pfdhfr* mutations in different areas has been provided in supplementary Additional file 1: Tables S1. The haplotype diversity (Hd) = 0.611 was estimated in *pfdhfr* gene with variance of haplotype diversity: 0.00081 and standard deviation of haplotype diversity: 0.028. The nucleotide diversity per site estimated was Pi = 0.00183, with sampling variance of Pi: 0.0000005 and standard deviation of Pi: 0.00067. The average number of nucleotide differences was k = 0.920,theta (per sequence) from S, Theta-W = 0.668 and theta (per site) from S, Theta-W = 0.00133.For *dhfr* gene analysis Tajima's D was 0.65908 (Not significant, P > 0.10). Fu and Li's D* test and Fu and Li's F* test statistic also showed not significant at P > 0.10 with a value of 0.87589 and 0.95422 respectively. The value of Fu’s Fs statistic = 0.001 and Strobeck’s S statistic = 0.685.

**Table 2:** Regional distribution of pfdhfr mutations

		*pfdhfr* genotypes											
State	N	N51I			C59R			S108N			I164L		
		N	I	N + I	C	R	C + R	S	N	S + N	I	L	I + L
High transmission areas													
Jharkhand	84	84	-	-	8	46	30	3	55	26	76	4	4
Odisha	70	67	2	1	22	40	8	21	43	6	66	1	3
Total	154	151 (98.05 %)	2 (1.3 %)	1 (0.65 %)	30 (19.5 %)	86 (55.8 %)	38 (24.7 %)	24 (15.6 %)	98 (63.6 %)	32 (20.8 %)	142 (92.2 %)	5 (3.2 %)	7 (4.5 %)
Low transmission areas													
Andhra Pradesh	32	32	-	-	17	8	7	14	10	8	32	-	-
Uttar Pradesh	31	31	-	-	4	23	4	-	31	-	31	-	-
Total	63	63 (100 %)	-	-	21 (33.3 %)	31 (49.2 %)	11 (17.5 %)	14 22.2 %)	41 (65.08 %)	8 (12.7 %)	63 (100 %)	-	-

**Table 3:** Regional distribution of *pfdhps* mutations

State	N	*pfdhps* genotypes											
		S436A			A437G			K540E			A581G		
		S	A	S + A	A	G	A + G	K	E	K + E	A	G	A + G
High transmission areas													
Jharkhand	84	76	6	2	55	25	4	76	6	2	64	17	3
Odisha	70	58	10	2	58	11	1	57	10	3	70	-	-
Total	154	134 (87.0 %)	16 (10.4 %)	4 (2.6 %)	113 (73.4 %)	36 (23.4 %)	5 (3.2 %)	133 (86.4 %)	16 (10.4 %)	5 (3.2 %)	134 (87.0 %)	17 (11.0 %)	3 (1.9 %)
Low transmission areas													
Andhra Pradesh	32	32	-	-	32	-	-	32	-	-	32	-	-
Uttar Pradesh	31	31	-	-	31	-	-	31	-	-	31	-	-
Total	63	63 (100 %)	-	-	63 (100 %)	-	-	63 (100 %)	-	-	63 (100 %)	-	-

While in *pfdhps* gene, wild-type alleles were predominant (79.26 %, *n* = 172) at all the codons (Table 2). Pure mutant alleles were observed in 7.37 (*n* = 16), 16.58 (*n* = 36), 7.83 (*n* = 17) and 7.83 % (*n* = 17) at codons 436, 437, 540 and 581 respectively. However, mixed mutant alleles were also prevailed in 1.84, 2.30, 2.30 and 1.38 % at codons 436, 437, 540 and 581 respectively. Year-wise breakup of *pfdhps* mutations in different sites has been provided in supplementary Additional file 2: Tables S2. The haplotype diversity Hd = 0.335 was estimated in *pfdhps* gene with variance of haplotype diversity = 0.00161 and standard deviation of haplotype diversity = 0.040.The nucleotide diversity per site estimated was Pi = 0.00133, sampling variance of Pi = 0.0000004and standard deviation of Pi = 0.00059. The average number of nucleotide differences was k = 0.756, theta (per sequence) from S, Theta-W = 0.673 and theta (per site) from S, Theta-W = 0.00118. For *dhps* gene analysis Tajima's D was 0.21856 (Not significant, P > 0.10). The Fu and Li's D* test statistic = 0.87896 and Fu and Li's F* test statistic = 0.78220 were also not statistical significant (P > 0.10). The Fu's Fs statistic = −2.372 and Strobeck’s S statistic = 0.965

### Mutant genotype status of *pfdhfr* and *pfdhps* genes

In *pfdhfr* gene, pure wild genotype ANCSI was found in 17.51 % (*n* = 38) isolates. Prevalence of single mutant (ANC***N***I), double mutant (AN***RN***I) genotypes were observed in 2.76 (*n* = 6) and 46.08 % (*n* = 100) isolates while mixed single mutant genotype (ANCN*I) and mixed double mutant genotypes (ANR*N*I) were observed in 2.76 (*n* = 6) and 23.50 % (*n* = 51) isolates respectively. Pure triple mutant genotypes were two type AN***RNL*** (3.22) and A***IRN***I (0.92 %) while mixed triple mutant genotypes ANRNL* (2.30) and AI*RNI (0.46 %). No quadruple mutant genotypes were found in *pfdhfr* gene.

In case of *pfdhps* gene, majority of isolates (79.26 %) were found pure wild genotype SAKAA. S***G***KAA (1.84 %), ***A***AKAA (0.46 %) and SA***E***AA genotype (0.46 %) are different pure single mutant genotypes, while SAE*AA (0.92 %) is mixed single mutant genotype. Prevalence of pure double mutant genotype (S***G***K***G***A) and triple mutant genotype (***AGE***AA) were (7.83) and (6.91 %) respectively, while mixed double mutant (SG*KG*A) and triple mutant (A*GEAA) were (0.92) and (1.38 %) respectively. Only one isolate showed mixed quadruple mutant genotype i.e. A*GEGA.

### Two-locus mutation status of *pfdhfr* and *pfdhps* genes

A total of 13 different *pfdhfr-pfdhps* two locus genotypes were observed among 217 isolates which are presented in Table 4. Wild-type two locus genotypes were observed in 17.51 % (*n* = 38) isolates. Mutant two locus genotypes were observed in 82.48 % (*n* = 179) isolates. Out of that, 62.6 % (*n* = 136) isolates showed mutant *pfdhfr* in the two-locus combination. The majority of isolates (115 of 136) had double mutant *pfdhfr* (GEN3) while single and triple mutant *pfdhfr* were found in 12 (GEN2) and 9 (GEN7 and GEN8 ) isolates respectively. Mutant genotype for both genes in the two locus combination was observed in 19.81 % (*n* = 43) isolates. The two locus combined mutant genotypes were categorized into various levels of clinical resistance as described earlier [18], which was based on number of mutations observed in the combination (Table 4). The category "S/RI" representing emergence of SP resistance was observed as genotype GEN2 and GEN3 in 58.52 % (*n* = 127) isolates. The category "RI" suggested low level of resistance against SP treatment was observed as genotype GEN4-6 in 2.30 % (*n* = 5) isolates. The category "RI/RII" suggested for high level of resistance against SP treatment was observed as genotype GEN7 in 1.38 % (*n* = 3) isolates. The category "RII" with quadruple mutation in two locus combination suggested for higher level of resistance against SP treatment was observed as genotype GEN8-10 in 11.52 % (*n* = 25) isolates. The category "RII/RIII" with quintuple mutation in two-locus combination also suggested for higher level of resistance against SP treatment was observed as genotype GEN11 in 5.53 % (*n* = 12) isolates. The category "RIII" with sextuple mutation in two locus combination suggested for level of resistance that can lead to total failure against SP treatment was observed as genotype GEN12-13 in 3.22 % (*n* = 7) isolates.

**Table 4:** Spatial distribution of *pfdhfr* and *pfdhps* allele combinations

Genotypes		*pfdhfr-pfdhps* allele combination	Resistance level^b^	Jharkhand (n=84)	Odisha (n=70)	Andhra Pradesh (n=32)	Uttar Pradesh (n=31)	Total (n=217)
Wild genotype								
	GEN 1	ANCSI-SAKAA	S	3 (3.57)	21 (30.00)	14 (43.75)		38 (17.51)
Mutant genotypes								
	GEN 2	ANC*N* ^a^I-SAKAA	S/RI	4 (4.76)	1 (1.43)	3 (9.38)	4 (12.90)	12 (5.53)
	GEN 3	AN*R* ^a^ *N* ^a^I-SAKAA	S/RI	45 (53.57)	28 (41.43)	15 (46.88)	27 (87.10)	115 (52.99)
	GEN 4	AN*RN*I-S*G*KAA	RI	1 (1.19)	1 (1.43)			2 (0.92)
	GEN 5	AN*R* ^a^ *N*I-*A*AKAA	RI		1 (1.43)			1 (0.46)
	GEN 6	AN*R* ^a^ *N* ^a^I-SA*E* ^a^AA	RI		2 (2.86)			2 (0.92)
	GEN 7	A*I* ^a^ *RN*I-SAKAA	RI/RII		3 (4.29)			3 (1.38)
	GEN 8	AN*RNL* ^a^-SAKAA	RII	4 (4.76)	2 (2.86)			6 (2.76)
	GEN 9	AN*R* ^a^ *N* ^a^I-S*G* ^a^K*G* ^a^A	RII	18 (21.42)				18(8.29)
	GEN 10	AN*RNL*-S*G* ^a^K*G* ^a^A	RII	1 (1.19)				1 (0.46)
	GEN 11	AN*RN*I-*A* ^a^ *GE*AA	RII/RIII	4 (4.76)	8 (11.42)			12 (5.52)
	GEN 12	AN*R* ^a^ *N* ^a^I-*A* ^a^ *G* ^a^ *E* ^a^ *G* ^a^A	RIII	1 (1.19)				1 (0.46)
	GEN 13	AN*R* ^a^ *N* ^a^ *L* ^a^-*A* ^a^ *G* ^a^ *E* ^a^AA	RIII	3 (3.57)	3 (4.29)			6 (2.76)

^a^mixed (wild + mutant)

^b^resistance level classified as per Ahmed *et al.* [18]

### Spatial distribution of mutations in *pfdhfr* and *pfdhps* genes

The wild-type ANCSI was observed at all study sites except Uttar Pradesh. Double mutant AN***RN***I allele in *pfdhfr* gene was prevalent in all four states Uttar Pradesh (87 %, *n* = 31), Jharkhand (83.33 %, *n* = 84), Odisha (57.14, *n* = 70) and Andhra Pradesh (46.87 %, *n* = 32). Triple mutants AN***RNL*** and A***IRN***I allele was observed in (5.71, *n* = 70) and (4.28 %, *n* = 70) isolates respectively at Odisha. Isolates from Jharkhand also showed AN***RNL*** in (9.52 %, *n* = 84) isolates. Thus mutation 164***L*** associated with higher level of drug resistance was present among isolates from Jharkhand (9.52) and Odisha (5.71 %), which are high transmission regions but not detected in Uttar Pradesh and Andhra Pradesh isolates. Mutation at codon 51 occurred rarely at Odisha (*n* = 3).

The wild-type SAKAA in *pfdhps* gene was prevalent at Jharkhand (65.47 %, *n* = 55) and Odisha (77.14 %, *n* = 54). No mutant *pfdhps* gene was observed at Andhra Pradesh and Uttar Pradesh. Single key mutation at position 437 (i.e. S***G***KAA) occurred rarely at Jharkhand (*n* = 2). While Odisha showed single mutant alleles at different positions which were at codon 436 (i.e. ***A***AKAA, *n* = 1) at key codon 437 (i.e. S***G***KAA, *n* = 1) and at codon 540 (i.e. SA***E***AA, *n* = 2). Mutation at codon position 581 was existed only in Jharkhand isolates with prevalence 23.80 % (*n* = 20). Double mutant allele S***G***K***G***A (23.80 %, *n* = 84) was observed only in Jharkhand isolates. Thus, Jharkhand and Odisha isolates showed mutations at all codons but with a varied rate. Triple mutant allele ***AGE***AA was observed in isolates from Odisha (15.71 %, *n* = 70) and Jharkhand (8.33 %, *n* = 84). Only one isolates from Jharkhand showed highly resistant quadruple mutant i.e. ***AGEG***A. No isolate was found to contain S436F and A613S/T mutations.

Uttar Pradesh and Andhra Pradesh showed the two-locus genotype GEN1 categorized as susceptible "S" and genotype GEN2-3 categorized as "S/RI" only. Whereas, Jharkhand and Odisha showed various genotypes categorized for susceptible (S), emerging resistance (S/RI, RI) and also high resistance (RII, RIII). In Jharkhand isolates, double mutant genotype (GEN3) was most prevalent in 53.57 % isolates, whereas the prevalence of triple mutant (GEN4, 8) and quadruple mutant (GEN9) genotypes were 5.95 and 21.42 % respectively. Highly resistant quintuple mutant genotype (GEN10-11) and sextuple mutant genotype (GEN12-13) were observed in five and four isolates respectively. In Odisha 30 % isolates showed wild-type genotype (GEN1). Like Jharkhand, Odisha also showed prevalence of double mutant genotype (GEN3) i.e. 41.43 % as compared to other mutant genotype. Prevalence of triple (GEN4-8), quintuple (GEN11) and sextuple mutant genotype (GEN12-13) was 12.85, 11.43 and 4.29 % respectively. Quadruple mutant genotype was absent in Odisha.

## Discussion

Currently, combination of fast and long acting antimalarial drugs is recommended as an ideal approach over the use of single antimalarial drug [3]. Optimizing the choice of long acting partner antimalarial drug in ACT is important challenge to be addressed in successful malaria treatment programme [33]. Presence of resistant parasites against the long acting antimalarial used in ACT can hamper the treatment efficacy and can also lead to emergence of artemisinin resistant parasite [7]. All malaria endemic parts of India experienced mutant parasites conferring resistance to all conventional antimalarial drugs like CQ, SP and thus there was country wide adoption of AS + SP as ACT in year 2010 [5]. However, resistance to SP had been well documented from northeastern part of India, which led to use of AS + lumefantrine as first-line malaria treatment in these parts of country since year 2013[7]. Northeast region of India has already been documented as important route for invasion of parasite bearing resistant genotypes against many antimalarials and proved its potential to be a focus for spread of resistant parasite to other parts of country [20]. Thus, monitoring of mutation status of partner SP is important for better management of antimalarial policy. Here, mutation status of *pfdhfr* and *pfdhps* gene responsible for resistance against SP was evaluated for isolates from four different geographic areas.

The study showed 17.51 wild-type *pfdhfr* gene and 79.26 % wild-type *pfdhps* gene. Higher number of mutant *pfdhfr* gene was observed in comparison to *pfdhps* gene at all the study sites infer development of mutations occurred first in *pfdhfr* gene and then in *pfdhps* gene under selective drug (SP) pressure. The prevalence of double mutant (AN***RN***I) in *pfdhfr* gene and wild-type *pfdhps* gene at all the study sites corroborated earlier reports of predominant presence for the same [23]. However, single mutant ANC***N***I, triple mutants (AN***RNL*** or A***IRN***I) in *pfdhfr* gene and single mutants (S***G***KAA, ***A***AKAA and SA***E***AA), double mutant S***G***K***G***A, triple mutant ***AGE***AA, quadruple mutant ***AGEG***A were also observed. Single or double *pfdhfr* mutations alone cannot cause SP treatment failure but the double *pfdhfr* mutations with a single or more mutation in *pfdhps* gene can cause various level of SP resistance [15]. In addition, triple mutant *pfdhfr* alone can cause various level of SP resistance. The DHFR-DHPS two locus mutations have importance to monitor as it can infer the clinical susceptibility of SP [15, 18]. This study observed 13 such two locus genotypes (GEN 1–13) within 217 isolates (Table 4). Out of all isolates, only 17.51 % were wild-type (GEN1). In total, double mutant genotype (GEN3) was observed in 52.99 % isolates and its predominance indicates continuous emergence of SP resistance in all study sites. The study sites include both high malaria transmission area (Odisha and Jharkhand) and low malaria transmission areas (Andhra Pradesh and Uttar Pradesh). Triple mutant genotype (GEN 4–8) that can confer high SP resistance were observed in Odisha and Jharkhand with prevalence of 12.85 and 5.95 % respectively, while quadruple mutant (GEN9) was found only in Jharkhand with 21.42 % prevalence. Isolates from high transmission areas also showed quintuple (Jharkhand = 5.95 %, Odisha = 11.43 %) and sextuple (Jharkhand = 4.76 %, Odisha = 4.29 %) mutant genotype. Quintuple and sextuple mutant genotypes associated with higher level of resistance to SP suggests selective drug pressure due to its use since a long period. Here, high transmission areas showed higher number of mixed mutations (both wild and mutant alleles) in both *pfdhfr* and *pfdhps* genes, which were possibly due to multi-clonal infection, as high recombination event is expected here, which in turn adding allelic variation. High genetic diversity at these high transmission areas in both gene under selection and neutral microsatellite markers were reported when compared to low transmission region of India [34, 35]. Higher genetic diversity and more fixation probability in genes responsible for various antimalarial resistance was observed earlier and suggested the role of malaria transmission intensity and drug exposure [36, 37]. The mutations 164**L** in *pfdhfr* and 437**G** and 540**E** mutations in *pfdhps* gene were reported to be responsible for therapeutic failure of SP [20, 22], and the same were observed in 9.52 and 34.52 % respectively in Jharkhand. In Odisha 4.28 % isolates showed another mutation 51**I** in *pfdhfr* gene which was responsible to accelerate the SP-resistance [15, 16]. In addition, these mutations are also part of two-locus genotypes (GEN 7–13) would be involved in clinical resistance against SP. In *pfdhps* gene, triple mutant ***AGE***AA was found in 15.71 % of Odisha isolates, while double mutant S***G***K***G***A is found only in 23.80 % of Jharkhand isolates. The prevalence of mutants found here in high transmission areas are similar to reported earlier from northeastern region [20, 22], however the prevalence of mutant two locus genotypes were not similar. Mutations like S436**F**, A613**T**/**S** was not observed in this study.

In contrast, the low transmission areas (Uttar Pradesh and Andhra Pradesh) showed single mutation (11.11 %) at codon positions 108, double mutations (66.67 %) at codon positions 59 and 108 in *pfdhfr* gene, while no isolate showed the N51**I** and I164**L** mutations associated with SP treatment failure. Thus the triple and quadruple mutations were not observed in *pfdhfr* gene. In case of *pfdhps* all isolates were wild-types, which infers *P. falciparum* population in these regions were susceptible to SP treatment and resistance could be in developing state. The low transmission areas showed mutations similar to earlier reports of similar single and double mutations from Uttar Pradesh and Delhi in year 2001, which suggested higher susceptibility for SP was maintained due to higher clonal populations in these regions [18]. In addition, *P. vivax* is prevalent in Uttar Pradesh and chloroquine is still effectively used as antimalarial treatment against *P. vivax* in India, which could provide selection pressure on gene responsible for chloroquine resistance in *P. falciparum* [34]. Thus no or low selection pressure of antifolate drugs in *P. falciparum* was predicted in these *P. vivax* prevalent areas as misdiagnosed of mixed infection cases was more exposed to chloroquine in comparison to antifolate drug .

## Conclusion

In conclusion, the present findings suggest that SP can be effective for the treatment of uncomplicated falciparum malaria as a partner drug of ACT in Andhra Pradesh and Uttar Pradesh (low transmission areas). In Jharkhand and Odisha (high tra**n**smission area), results suggest that mutation rate will increase continuously due to continued drug pressure a*nd mala*ria transmission, which in turn will lead to SP treatment failure in near future*,* as reported in northeastern parts of India. Continuous molecular surveillance of partner drug (SP) in these high transmission areas is required to maintain an effective drug policy.

## Additional files

Additional file 1: Table S1. Spatial and temporal distribution of *P. falciparum dhfr* point mutations among Indian isolates. (DOC 57 kb) Additional file 2: Table S2. Spatial and temporal distribution of *P. falciparum dhps* point mutations among Indian isolates. (DOC 54 kb)
