# Supplementary material for: Polymorphism in drug resistance genes dihydrofolate reductase and dihydropteroate synthase in Plasmodium falciparum in some states of India
Source: Parasit Vectors. 2015 Sep 17;8:471. doi: 10.1186/s13071-015-1080-2 (PMC4574150; doi:10.1186/s13071-015-1080-2)
Supplement: Additional file 1: Table S1. — Spatial and temporal distribution of P. falciparum dhfr point mutations among Indian isolates. (DOC 57 kb) [file 13071_2015_1080_MOESM1_ESM.doc]

**Table S1:** Spatial and temporal distribution of *P. falciparum dhfr* point mutations among Indian isolates.

|  | | Codons & genotypes (*pfdhfr*) | | | | | | | | | | | |
| --- | --- | --- | --- | --- | --- | --- | --- | --- | --- | --- | --- | --- | --- |
|  | | N51I | | | C59R | | | S108N | | | I164L | | |
| State | N | N | I | N+I | C | R | C+R | S | N | S+N | I | L | I+L |
| Jharkhand |  |  |  |  |  |  |  |  |  |  |  |  |  |
| 2006 | 20 | 20 | - | - | - | 8 | 12 | - | 6 | 14 | 18 | - | 2 |
| 2007 | 4 | 4 | - | - | - | 3 | 1 | - | 3 | 1 | 3 | - | 1 |
| 2008 | 23 | 23 | - | - | 4 | 17 | 2 | 1 | 18 | 4 | 20 | 2 | 1 |
| 2009 | 14 | 14 | - | - | - | 9 | 5 | - | 12 | 2 | 13 | 1 | - |
| 2010 | 14 | 14 | - | - | - | 9 | 5 | - | 14 | - | 13 | 1 | - |
| 2011 | 9 | 9 | - | - | 4 | - | 5 | 2 | 2 | 5 | 9 | - | - |
| Total | 84 | 84 | - | - | 8 | 46 | 30 | 3 | 55 | 26 | 76 | 4 | 4 |
| Odisha |  |  |  |  |  |  |  |  |  |  |  |  |  |
| 2008 | 20 | 20 | - | - | 10 | 6 | 4 | 9 | 8 | 3 | 20 | - | - |
| 2010 | 34 | 33 | 1 | - | 7 | 23 | 3 | 8 | 24 | 2 | 31 | 1 | 2 |
| 2011 | 10 | 8 | 1 | 1 | 1 | 8 | 1 | 1 | 8 | 1 | 9 | - | 1 |
| 2012 | 6 | 6 | - | - | 3 | 3 | - | 3 | 3 | - | 6 | - | - |
| Total | 70 | 67 | 2 | 1 | 22 | 40 | 8 | 21 | 43 | 6 | 66 | 1 | 3 |
| Andhra Pradesh |  |  |  |  |  |  |  |  |  |  |  |  |  |
| 2011 | 32 | 32 | - | - | 17 | 8 | 7 | 14 | 10 | 8 | 32 | - | - |
| Uttar Pradesh |  |  |  |  |  |  |  |  |  |  |  |  |  |
| 2011 | 17 | 17 | - | - | 2 | 13 | 2 | - | 17 | - | 17 | - | - |
| 2012 | 14 | 14 | - | - | 2 | 10 | 2 | - | 14 | - | 14 | - | - |
| Total | 31 | 31 | - | - | 4 | 23 | 4 | - | 31 | - | 31 | - | - |
