# Supplementary material for: Polymorphism in drug resistance genes dihydrofolate reductase and dihydropteroate synthase in Plasmodium falciparum in some states of India
Source: Parasit Vectors. 2015 Sep 17;8:471. doi: 10.1186/s13071-015-1080-2 (PMC4574150; doi:10.1186/s13071-015-1080-2)
Supplement: Additional file 2: Table S2. — Spatial and temporal distribution of P. falciparum dhps point mutations among Indian isolates. (DOC 54 kb) [file 13071_2015_1080_MOESM2_ESM.doc]

- **Table S2:** Spatial and temporal distribution of *P. falciparum* *dhps* point mutations among Indian isolates.

| State | Codons & genotypes (*pfdhps* gene) | | | | | | | | | | | | | |
| --- | --- | --- | --- | --- | --- | --- | --- | --- | --- | --- | --- | --- | --- | --- |
|  |  | | S436A | | | A437G | | | K540E | | | A581G | | |
|  | Year | n | S | A | S+A | A | G | A+G | K | E | K+E | A | G | A+G |
| Jharkhand | 2006 | 20 | 19 | 1 | - | 15 | 5 | - | 19 | 1 | - | 16 | 4 | - |
|  | 2007 | 4 | 3 | - | 1 | - | 3 | 1 | 3 | - | 1 | 1 | 2 | 1 |
|  | 2008 | 23 | 22 | 1 | - | 17 | 4 | 2 | 22 | 1 | - | 18 | 3 | 2 |
|  | 2009 | 14 | 12 | 2 | - | 7 | 7 | - | 12 | 2 | - | 10 | 4 | - |
|  | 2010 | 14 | 11 | 2 | 1 | 8 | 5 | 1 | 11 | 2 | 1 | 11 | 3 | - |
|  | 2011 | 9 | 9 | - | - | 8 | 1 | - | 9 | - | - | 8 | 1 | - |
| Total |  | 84 | 76 | 6 | 2 | 55 | 25 | 4 | 76 | 6 | 2 | 64 | 17 | 3 |
| Odisha | 2008 | 20 | 20 | - | - | 20 | - | - | 20 | - | - | 20 | - | - |
|  | 2010 | 34 | 28 | 5 | 1 | 28 | 6 | - | 27 | 5 | 2 | 34 | - | - |
|  | 2011 | 10 | 4 | 5 | 1 | 4 | 5 | 1 | 4 | 5 | 1 | 10 | - | - |
|  | 2012 | 6 | 6 | - | - | 6 | - | - | 6 | - | - | 6 | - | - |
| Total |  | 70 | 58 | 10 | 2 | 58 | 11 | 1 | 57 | 10 | 3 | 70 | - | - |
| Andhra Pradesh | 2011 | 32 | 32 | - | - | 32 | - | - | 32 | - | - | 32 | - | - |
| Uttar Pradesh | 2011 | 17 | 17 | - | - | 17 | - | - | 17 | - | - | 17 | - | - |
|  | 2012 | 14 | 14 | - | - | 14 | - | - | 14 | - | - | 14 | - | - |
| Total |  | 31 | 31 | - | - | 31 | - | - | 31 | - | - | 31 | - | - |
